# The incidence of VP shunt infection in a middle-income nation: a retrospective analysis of a pediatric population

**DOI:** 10.3389/fsurg.2023.1304105

**Published:** 2023-12-20

**Authors:** Dwayne Campbell, Shane Sinclair, Dwaine Cooke, Dwight Webster, Marvin Reid

**Affiliations:** ^1^ Department of Neurosurgery, Kingston Public Hospital, Kingston, Jamaica; ^2^ Graduate Studies and Research, University of the West Indies, Mona, Jamaica

**Keywords:** ventriculoperitoneal shunt infection, VPS, shunt infection risk, Jamaica shunt, Bustamante hospital, pediatric

## Abstract

**Objective:**

To investigate the incidence of infection after ventriculoperitoneal shunt (VPS) insertion at the Bustamante Hospital for Children (BHC), Jamaica, West Indies.

**Method:**

Of the 178 patients managed by the Neurosurgery team at BHC, who underwent surgery between 2010 and 2016, 122 patients were subjected to the cerebrospinal fluid (CSF) diversion procedure through a VPS placement. The patients excluded from this study included those with a VPS placed at another institution or one placed prior to the study period. There is a notable transition that saw a switch from the use of the Codman uni-port to Medtronic shunts in 2014–2015, which initiated the process of reuse of shunt passers. Clinical data were retrospectively collected from operating theater logs and available manual health records.

**Results:**

Over the 7-year study period of the 122 first-time shunt placements done, 17 patients (13.9%) had positive CSF cultures, with an additional six (4%) having CSF pleocytosis with negative cultures. The most common isolate was the *Staphylococcus* species, occurring in 60% of VPS infections. The median time to shunt infection was 2 months. Of the 72 Codman shunts placed, six became infected, and 21.7% (10 of 46) of the Medtronic shunts became infected.

**Conclusion:**

The rate of incidence of VPS infection was 13.9% for the period between 2010 and 2016, with most infections occurring after 2014. The major causative agent was *Staphylococcus* species at 60% within a median 2 months of surgery. Overall, this compares well with data reported in the literature.

## Introduction

Hydrocephalus refers to the accumulation of cerebrospinal fluid (CSF) in the ventricular spaces and cisterns, arising from a disturbance in its formation, absorption, or flow. It is a common condition of childhood associated with disease processes such as tumors, head injury, intracranial hemorrhage, spina bifida, and meningitis (1). In the pediatric population, it is commonly treated surgically using a ventriculoperitoneal shunt (VPS), a device usually placed in the lateral ventricle traveling subcutaneously and draining into the peritoneal cavity.

A VPS infection is defined as a positive CSF culture in association with CSF pleocytosis, fever, neurologic symptoms, and signs of shunt malfunction (2, 3). This will be extended to include a positive blood culture with symptoms of altered neurology, fever, peritonism, vomiting, headache, and seizures (new onset).

Hydrocephalus may be treated with the CSF diversion procedure; this may be in the form of endoscopic third ventriculostomy (ETV) recommended locally by Crandon et al. (4) as the treatment for obstructive hydrocephalus. VPS, however, can be used to treat both communicating and non-communicating hydrocephalus.

At the Bustamante Hospital for Children (BHC), Jamaica, shunting is the procedure of choice. Initially, Codman branded shunts were utilized, and a shift from Codman to Medtronic shunts occurred for reasons that were multifactorial. Medtronic shunts had detachable parts, which made revision of a component simpler, which, in turn, made it possible to reduce the extent of surgery done. In addition, Medtronic shunts cost less compared with Codman shunts. Against this background, this study aims to examine the rate of infection in the two types of shunting procedures and the overall incidence of shunt infection.

## Methods

### Patient population

The BHC is the only specialist pediatric hospital in the English-speaking Caribbean, catering to patients between birth and 12 years of age. It is located in Jamaica, which is classified as a middle-income country within Latin America and the Caribbean Region by the United Nations (5). A retrospective review of all the VPSs placed at the BHC by the Department of Neurosurgery during the period between 2010 and 2016 was performed. Ethical approval was obtained from both the Ethical and Research Committee of the South East Regional Health Authority (SERHA) and the BHC.

A VPS infection was defined as a CSF-positive culture or as a CSF pleocytosis (WBC >9), with symptoms such as fever, vomiting, and abdominal pain, with positive blood culture. Patients were excluded if the VPS was placed secondary to an infective process (e.g., post-meningitis), if the shunt was not initially placed at the BHC, and if the patient had already been diagnosed but no documentation of CSF culture or pleocytosis and other clinical symptoms was available.

During the study period, shunting procedures were done on 178 patients at the BHC, but 56 were excluded from the study. Of the remaining 122 investigated at the 1-year follow-up after the surgery, 10 patients had died and 6 patients lost to follow-up, with only 106 confirmed to be alive at the end of the period. Shunts were placed via Keen's point as described by Morone et al. (6); with the majority (85%) (104) being on the right. All patients underwent both preoperative and postoperative imaging (computed tomography or magnetic resonance imaging), which allows for a comparison of ventricular size and radiological confirmation of placement.

Of the 122 patients included, the primary diagnosis at the time of shunt placement was hydrocephalus (not specified) (48), followed by cranial malformations (30) and neural tube defects (20), as listed in Table 1.

**Table 1:** Primary diagnosis at the time of VPS placement.

Diagnosis	Number (%)
Tumors	18 (14.7)
Cranial congenital malformations	30 (24.6)
Hemorrhage	6 (4.9)
Hydrocephalus (not specified)	48 (39.3)
Neural tube defects	20 (16.4)

### Shunting

VPS placement at the BHC was done initially with the non-antibiotic-impregnated, non-programmable Codman uni-shunts; these were packaged with their own shunt passers, which made the equipment single use. However, between 2014 and 2015, there was a transition to the non-antibiotic-impregnated, non-programmable Medtronic shunts, which triggered the beginning of the era of reuse of shunt passers. These passers were manually cleaned and sterilized in an autoclave.

Shunts were placed only in clinically and laboratory-proven non-infected patients, under general anesthesia. Standard pressure valves were used, varying them from low to high pressure. In all, 46 Medtronic shunts and 72 Codman uni-shunts were covered by the study, with three other shunts of brands that were undocumented and one was obtained from Integra LifeSciences Corp, Princeton, NJ, USA (Now Integra)—OSV ii. Of these, 98 (83.1%) were of medium pressure, 15 (12.7%) of high pressure, and three (2.5%) of low pressure. The pressure of the shunt was unknown in six cases. Preoperative antibiotics were administered to all those who underwent the shunt procedures, with cephalosporin being the most used (ceftriaxone in 117 patients and cefuroxime in four) and gentamicin in one patient. In terms of gender, of the 122 patients, 53 (43.4%) were females and 69 (56.6%) were males, with a median gestational age and birth weight of 38 weeks and 2.95 kg, respectively. At the time of surgery, the median age and weight were 4 months and 6.27 kg, respectively.

## Results

In the analysis of the data collected, 122 observations were made of eligible patients. As mentioned previously, of these, 10 patients died, of which three had no exit date, and six patients were lost to follow-up, of which four had no exit date. The data set (Table 2) examining those alive at 1 year showed a total of 106 patients; however, for the analysis, those without exit dates were not considered.

**Table 2:** One-year outcome post VPS.

One-year outcome	Frequency	Percent
Alive	106	92.17
Died before 1 year	7	6.09
Lost to follow-up prior to 1 year	2	1.74
Total	115	100.00

Table 3 provides the possible factors associated with the development of VPS infection, which were determined considering those related to both the patient and the surgeon. Most factors were noted to be insignificant with a *p*-value >0.05. Birth weight, gestational age, and length of stay in hospital after shunting were noted to be significant at *p*-values <0.05 in the development of a shunt infection. The median age and weight varied with gender, being 3–5 months and 5–6 kg, respectively. The length of surgery, while established as a risk factor in the literature, did not impact this study, as the median operative time ranged between 58 and 60 and 60 and 65 min, respectively, in the infected and non-infected groups.

**Table 3:** Factors affecting the development of shunt infections.

Clinical characteristics	Non-infected		Infected		*P*-value
	Female	Male	Female	Male	
	Median		Median		
Age (months)	4	3	5	3	0.419
BW (kg)	2.9	3	2.1	2.9	0.006
Weight (kg)	6.4	6.6	6	5.2	0.789
GA (weeks)	37	38	32	39	0.035
Op time (min)	58	60	65	60	0.258
Hospital stay (months)	0.3	0.5	0.4	0.4	0.032

BW, birthweight; GA, gestational age; Op, operative; min, minimum; max, maximum.

Neither the type or brand of shunt used nor the level of the lead surgeon, that is, whether a senior resident or a consultant, with a median time of 63.5 and 52 min, respectively, had any impact on the development of shunt infection. Notably, nine of the 17 shunt infections occurred when the lead surgeon was a resident.

The most common isolate was *Staphylococci* species (*S. aureus*, *S. epidermidis*, *S. saprophyticus*) in 50% of the cases, followed by no identified organism in 21% of cases, although other clinical and biochemical markers persisted. Approximately 55%–60% of cultures had sensitivity to vancomycin and gentamicin, followed by trimethoprim/sulfamethoxazole and clindamycin at 30%–35%. Empiric antibiotic included Rocephin (Ceftriaxone) in 100% of cases; however plating with isolate was done in only one case. Other empiric antibiotics included Augmentin and gentamicin, with the latter being the most common. Penicillin resistance was noted in 50% of patients, Augment was plated in only two patients, with one patient being resistant and the other sensitive. Metronidazole was used empirically in 30% of patients and plated only in one patient.

Over the period of the study, the incidence of VPS infection within 1 year of placement occurred in 17 out of the 122 patients at a rate of 13.9%, with a median time of 2 months after the procedure and with 21.7% of Medtronic shunts and 8% of the Codman shunts becoming infected.

## Discussion

The placement of VPSs significantly improved the management outcome of hydrocephalus; the shunts, however, come with several complications, such as infection, obstruction, abdominal pseudocyst, bowel perforation, over drainage, and subdural hematoma (SDH) (7). Shunting procedures are variable, with common options being Kocher's, Keen's, or Frazier's points, with each having unique advantages and disadvantages (6). Having such a variety of techniques is essential in management as it allows for a consideration of the type of hydrocephalus and other patient factors (8). It was noted that while Kocher's point is the most common point for the ventricular tap, it is a difficult procedure due to the rotated position of the head during shunting. While Frazier's point provides an easier entry to the body of the lateral ventricle, there is a risk of damage to the superior sagittal and transverse sinus or visual cortex. At our institution, the preference is for Keen's point by senior neurosurgeons and the Chief of Neurosurgery, which is primarily used during the training of residents. This procedure is done by free hand, as image guidance [the gold standard as noted by Ganau et al. (9)] is not available to us. The success rate of freehand placement of ventricular catheter has been noted to be 44%–64% for Kocher's and 67%–71% with occipital and parieto-occipital approaches (8). Shunt malfunction most commonly arises from obstruction, followed by shunt infection. According to McGirt et al. (10), 30%–40% of all pediatric VPSs fail or malfunction within the first year, with centers having a revision to primary placement ratio of 3:1. In addition, it has been noted that many patients may have up to 14 revisions of shunt procedure in their lifetime.

The rate of incidence of shunt infection varies in the literature. Paff et al. (7) noted a rate of 8%–15%, while Bennett et al. (11) noted that the incidence rate ranged from 5% to 41% in various series, although the general range is 4%–17%. It is theorized (2) that biofilm-forming bacteria attach to the surface of the implanted device, forming a heterogeneous structure of the complex cellular matrix. What is of importance is that biofilms can evade the host immune system and are increasingly resistant to antibiotic therapy compared with planktonic forms (12).

A study published in the *Journal of Pediatric Neurosurgery* (13) examined the rate of VPS infection across pediatric hospitals in the United States. The study involved 7071 patients with uncomplicated shunt placement, followed up for 1 year; it was found that the average infection rates were 11.7% per patient and 7.2% per procedure. The factors that contributed to this infection rate (having a *p* < 0.05) included young age and female gender, the African American race, surgeon case volume, and hospital volume, and the protective factors included intracranial malignant lesions and head trauma. In a retrospective cohort study of 333 children at the Seoul National University Children’s Hospital, Korea, conducted between 2005 and 2011 (14), it was found that 10.5% developed a shunt infection. It was further noted that the median time interval was 1 month (range 6 days to 8 months) post placement, with the conclusion drawn that an independent risk factor for shunt infection was having the procedure within the first year of life, with a relative risk of 2.31.

With regard to the diagnosis of a shunt infection, while there has been reservations about shunt tapping, with some pointing to the potential risk of introducing pathogens during the tapping, most studies fail to provide conclusive evidence of the same. It has been shown, however, that 92%, 66.7%, and 30.4% of aspirated shunt fluid, lumbar puncture samples, and blood cultures, respectively, were positive for causative organisms, making shunt tapping the most efficient diagnostic tool (15). *Staphylococcus* species was noted in 53% of cases in a study by McGirt et al. (10). According to Paff et al. (7), shunt infections occur because of contamination of the device by skin flora (*S. epidermidis* and *S. aureus*), with symptoms presenting early (<14 days) postoperatively. The presence of Methicillin resistance ranges from as low as 10% (five cases of the 49 positive *Staphylococci* shunt infections) in the study done by McGirt et al. in the USA, to as high as 83% as shown by Lee et al. in South Korea.

Schoenbaum et al. (16) noted that infection rates were lower in patients who received antistaphylococcal antibiotics in the perioperative period. While perioperative antibiotics are helpful in reducing the risk of infection, the optimal agent is not known; this is attributed to the resistance pattern, limiting beta-lactam use, and the now growing concern of resistance rates, which limits the efficacy of vancomycin (17, 18). It was also found that although infection rates were lower after the instillation of intraventricular antibiotics during surgery, whatever infections occurred could not be specifically attributed to the intraventricular antibiotics (19). A potential option is immunotherapy or vaccines to targeted *Staphylococci* species; however, this has proved to be difficult due to the lack of protective immunity after natural Staphylococcal infection (20). It was further demonstrated in a retrospective study by Honeycutt et al. (21) that infection rates were lower when traffic during the operative time was reduced. Although it was demonstrated that the use of antibiotic-impregnated shunts helped successfully kill 100% adhered microbes in 48–52 h, it does not prevent adherence (22). However, some researchers (23, 24), found no significant reduction in the infection rates reported in retrospective studies nor in those reported in the clinical trials of conventional vs. antibiotic-impregnated devices. It is worth mentioning that in a meta-analysis of antibiotic-impregnated shunts (AISs) vs. the conventional shunt, it was found that the AIS was both protective and cost effective (25). The antibiotics of choice for empiric therapy according to the Infectious Disease Society of America are vancomycin and ceftazidime, cefepime, or meropenem (26); however, due to pharmacokenetic factors, bioavailability in the central nervous system (CNS) at therapeutic concentrations may be limiting, whereas intraventricular administration may help overcome these hurdles (10, 27, 28). Intraventricular administration of antibiotics, in addition to shunt removal and externalization of the ventricular drain, was found to cure 14 of the 15 children with shunt infections at the People’s Liberations Army (PLA) hospital in China (29).

The incidence of VPS infection within 1 year of placement is similar to that seen internationally. Despite the restriction in resources, a rate of 14% is noted, at a median interval of 2 months mainly associated with birth weight, length of stay in hospital, and gestational age. Although seen elsewhere, weight and age at the time of surgery seem to have had no impact on this patient population. Paff et al. (7) note that most shunt infections occur within <14 days of surgery, unlike in our setting, where most occur 2 months postoperatively. Notably, skin commensals, the organisms noted by Paff et al. (7), were the same ones reported in our patient population.

The rate of shunt infection was notably higher in the Medtronic group than in the Codman group, at 21% and 8%, respectively, with a *p*-value of 0.278, making it non-significant. This is similar to the findings of Woo et al. (30) who studied shunt placement between 2009 and 2011 at their institution. When the shunt brand was evaluated with respect to the incidence of infection, that is, Codman vs. Medtronic, it was found to be non-significant.

A recommendation can be made to plate cultures for ceftriaxone because it was the most used preoperative antibiotic. A switch to preoperative vancomycin and gentamicin may prove beneficial as 60% of shunt cultures were sensitive, which is in keeping with recommendations from the Infectious Disease Society of America (26).

## Conclusion

The median time to shunt infection was noted to be 2 months post procedure, at a rate of 13.9% of new shunts placed, with the time extending up to 1 year. While the causative organism in the infection varied, as did the antibiotic therapy, vancomycin and gentamicin covered up to 60% of patients, making a strong case for starting these antibiotics empirically.

## Data Availability

The raw data supporting the conclusions of this article will be made available by the authors without undue reservation.
